# Factors influencing adherence in Hepatitis-C infected patients: a systematic review

**DOI:** 10.1186/1471-2334-14-203

**Published:** 2014-04-15

**Authors:** Tim Mathes, Sunya-Lee Antoine, Dawid Pieper

**Affiliations:** 1 Institute for Research in Operative Medicine, Faculty of Health - School of Medicine, Witten/Herdecke University, Ostmerheimer Str. 200, Building 38, D- 51109 Cologne, Germany

**Keywords:** Medication adherence, Ribavirin, Hepatitis-C, Systematic review

## Abstract

**Background:**

Adherence is a crucial point for the successful treatment of a hepatitis-C virus infection. Studies have shown that especially adherence to ribavirin is important.

The objective of this systematic review was to identify factors that influence adherence in hepatitis-C infected patients taking regimes that containing ribavirin.

**Methods:**

A systematic literature search was performed in Medline and Embase in March 2014 without limits for publication date. Titles and abstracts and in case of relevance, full-texts were screened according to predefined inclusion criteria. The risk of bias was assessed. Both process steps were carried out independently by two reviewers. Relevant data on study characteristics and results were extracted in standardized tables by one reviewer and checked by a second. Data were synthesized in a narrative way using a standardized procedure.

**Results:**

Nine relevant studies were identified. The number of analyzed patients ranged between 12 and 5706 patients. The study quality was moderate. Especially the risk of bias regarding the measurement of influencing factors was mostly unclear.

“Psychiatric disorders” (N = 5) and having to take “higher doses of ribavirin” (N = 3) showed a negative influence on adherence. In contrast, a “HIV co-infection” (N = 2) and the “hemoglobin level” (N = 2) were associated with a positive influence on adherence. Furthermore, there is the tendency that male patients are more adherent than female patients (N = 6). “Alcohol consumption” (N = 2), “education”, “employment status”, “ethnic group“,”hepatitis-C virus RNA” (N = 4), “genotype” (N = 5), “metavir activity” (N = 1) and “weight” (N = 3) showed mostly no effect on adherence. Although, some studies showed statistically significant results for “age”, “drug use” , “genotype”, “medication dose interferon“, and “treatment experience” the effect is unclear because effect directions were partly conflicting.

The other factors were heterogeneous regarding the effect direction and/or statistical significance.

**Conclusion:**

There are some factors that seem to show an influence on adherence. However, due to the heterogeneity (e.g. patient characteristics, regimes, settings, countries) no general conclusions can be made. The results should rather be considered as indications for factors that can have an influence on adherence in hepatitis-C infected patients taking regimes that containing ribavirin.

## Background

In developed countries the prevalence of hepatitis-C virus infections ranges between 1.3 and 2.7% [1]. About 18.5% of hepatitis-C virus infected patients develop cirrhosis and 1.3% a hepatocellular carcinoma [2]. Prior research has shown that adherence, as “the extent to which a patient acts in accordance with the prescribed interval and dose of a dosing regimen” is a crucial point for successful treatment of a hepatitis-C virus infection [3-5]. Although new drugs for the treatment of Hepatitis-C entered the market, ribavirin is still important to reach sustained virologic response (SVR) [6]. Studies have shown that reducing the dose of ribavirin from ≥80% to ≤60% resulted in a decline of SVR form 21% to 11% (p ≤ 0.05). In contrast, reduced peg-interferon use was not associated with a decline in SVR [7]. Furthermore, research has shown that in the combination therapy for hepatitis-C with interferon and ribavirin, adherence is lower for ribavirin than for interferon [8].

Several factors (e.g. patient characteristics, treatment characteristics, disease characteristics, setting) exist that can potentially influence patient adherence. The factors can be grouped in the following five dimensions: social and economic, health care system, health condition, therapy and patient [9].

Systematic reviews for various indications have identified factors that can influence patient adherence [10-12]. For example, Bowry et al. [10] found poor knowledge, negative perceptions about medication, side effects and high medication costs to be predictive for non-adherence in patients taking cardiovascular medications. Jackson et al. [11] could not find a clear effect for any demographic, clinical, or treatment factors in patients with bowel disease. Verbrugghe et al. showed that younger age and side effects were the two predominate factors for adherence in patients taking oral anti-cancer agents [12]. But to the best of our knowledge there is no systematic review that investigates adherence influencing factors in hepatitis-C virus infected patients The objective of this systematic review was to identify factors that influence adherence in hepatitis-C infected patients prescribed with regimen that contain ribavirin.

## Methods

### Sources

This systematic review was prepared according to the standards of the recommendations for systematic reviews of prognostic factors and reported according to MOOSE [13,14]. A systematic literature search was performed in MEDLINE (via Pubmed) and Embase (via Embase) (TM). The search strategy combined various terms and medical subject headings related to adherence, hepatitis-C and ribavirin (the full search strategies for each database are available in Additional file 1). The search was performed on March 25th 2013. Study type, publication date and language were not limited in the electronic search strategy to maximize sensitivity.

### Study selection

To be eligible for this review the studies had to meet the following inclusion criteria:

1. Patients: Adult patients with hepatitis-C virus infection

2. Exposure: Potential adherence influencing factor/s (exposure [factor] is not controlled by the investigator, e.g. different dosages or therapies)

3. Medication: Regimes containing ribavirin

4. Outcome: Quantitative patient implementation adherence [15] measure (not persistence, not exclusively intentional-adherence measures)

5. Region: Study conducted in WHO- mortality Stratum A (very low child mortality and low adult mortality) [16]

6. Publication language: English or German

No exclusion criteria were applied.

Two reviewers independently performed the study selection according to the inclusion criteria in a two-step procedure (DP; SA, TM). Firstly, the titles and abstracts of all hits in the electronic databases were screened. Secondly, the full-texts of all potentially relevant articles were obtained and screened. Any differences between the reviewers were discussed until consensus was reached. In addition, the reference lists of all included publications were hand-searched and a Google Scholar search was performed to identify grey literature (TM). The authors were contacted in case of any missing information regarding the inclusion criteria (TM).

According to established recommendations a differentiation between initiation, implementation and discontinuation adherence should be made [15]. In clinical practice initiation and discontinuation of a medication regimen can be easily assessed. In contrast, implementation adherence is often not obvious. Furthermore, research indicates that the reasons for discontinuation and implementation adherence can differ [17]. Thus, it was decided to focus on implementation adherence. Non-adherence can be intentional (e.g. conscious decision not to take) or non-intentional (e.g. forgetting). Furthermore, it has been shown that non-adherence is mostly non-intentional and that there are different influencing factors [18,19]. Therefore, we excluded studies that only measured intentional non-adherence like surveys that gathered reasons for non-adherence stated by patients.

### Assessment of risk of bias

The risk of bias of included studies was assessed using the methodology checklist for prognostic studies provided by the National Institute for Health and Care Excellence (NICE) (evaluation questions for the instruments are available in Additional file 2) [20]. The study population was considered representative, if there were no specific inclusion criteria (e.g. psychiatric disorders) i.e. if the population was representative for the general hepatitis-C infected population in western countries. Confounding was rated appropriate if the potentially relevant factors were incorporated in the analysis. The risk of bias assessment was performed independently by two reviewers (TM, DP). Disagreements were resolved in a discussion or by involving a third person.

### Data extraction and synthesis

The data were extracted in pre-designed standardized tables. For each study the number of analyzed patients, the study inclusion criteria (demographic, socioeconomic, disease related, medication related), the country the study took place, the used adherence measure and mathematical operationalization, and the medications to which the adherence measure (ribavirin or ribavirin and interferon) refers to were extracted. With respect to the results, the influencing factor and the effect on adherence (effect direction or compared categories; effect size and measure) and the statistical significance (95%-CI or p-value) were extracted. All data in the tables on the influence refers to an increase of the respective factor, independent from whether the factor is positive (e.g. educational level) or negative (adverse events). Higher risk ratios (RR) and odds ratios (OR) mean lower adherence in the reference group. e.g. in a comparison of higher versus lower age, a higher OR indicates a higher adherence in the higher aged population. In case the studies used univariate as well as multivariate analysis methods, only the results of the multivariate analysis were extracted. Data extraction were performed by one reviewer (TM) and verified by a second (DP). If effect sizes and statistical significance were not reported in the publications, the OR with confidence intervals were calculated by the authors (Microsoft Excel 2010) using the double data entry method, provided that there were sufficient data (TM, DP).

A quantitative data synthesis using a meta-analysis was planned a priori but was not performed to avoid misleading results due to heterogeneity regarding the included patients, the adherence measurements and definitions/operationalization, the measurement of influencing factors, and the statistical analysis methods (e.g. adjustments, categorizations). Furthermore, in most studies there was a significant lack of reporting especially regarding values for not statistically significant results to allow a recalculation of data.

For all factors that were analyzed in at least two studies a summary estimation of the effect direction and the effect size was made. Two reviewers rated the evidence for an effect, considering the consistency of the effect direction (within and between studies), the effect size, the statistical significance, the sample size and the risk of bias of included studies that analyzed the respective factor (TM, DP). Discrepant ratings were discussed until consensus.

A p-level of < 0.05 was considered statistically significant.

## Results

**Figure 1:**
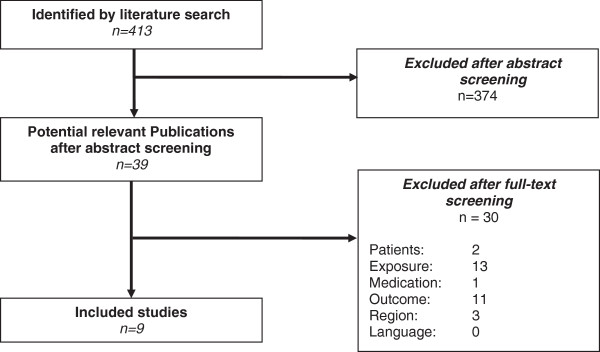
Flow-chart of study selection process.

The electronic literature search resulted in 413 hits. Thirty nine of the titles and abstracts seemed potentially relevant and full-text versions of the publications were screened. Nine studies met all inclusion criteria and were included in the systematic review [4,8,21-27]. The process of study selection and the reasons for exclusion are illustrated in the flowchart (see Figure 1).

The risk of bias was moderate. In particular the representativeness of the study sample and the consideration of confounders were insufficient in most studies. The studies that applied an adjusted analysis considered the potentially relevant confounders, i.e. the studies that were rated with minus were unadjusted. Furthermore, no statements on the measurement of the influencing factors and descriptions of the patients lost-to-follow-up could be found in many studies. The results of the risk of bias assessment for each study are presented in Table 1.

**Table 1:** Risk of bias of included studies

**Study**	**Representativeness of study sample**	**Loss to follow-ups**	**Measurement of prognostic factor**	**Measurement of outcome**	**Accounting for confounders**	**Statistical analysis**
Giannelli 2012 [21]	**-**	**+**	**+**	**?**	**?**	**?**
Lo Re [4]	**-**	**+**	**?**	**+**	**+**	**+**
Marcellin 2011 [8]	**+**	**+**	**?**	**+**	**+**	**+**
Martín-Santos 2008 [22]	**-**	**?**	**+**	**?**	**-**	**+**
Rodis [23]	**+**	**?**	**?**	**+**	**-**	**?**
Sola [24]	**-**	**+**	**?**	**+**	**+**	**+**
Sylvestre [25]	**-**	**+**	**+**	**+**	**-**	**+**
Tanioka [26]	**+**	**?**	**+**	**?**	**+**	**+**
Wagner [27]	**-**	**?**	**+**	**+**	**-**	**+**

**+** = yes; - = no; ? = unclear.

The number of analyzed patients ranged between 12 and 5706 patients. In two studies the study population was highly specified. Giannelli et al. [21], included only liver transplanted patients and Lo Re exclusively US Veterans [4]. Except the study by Marcellin et al. [8], all studies were performed in hospitals and medical centers. To measure adherence either pill counts or self-reports were applied. In two studies the analysis was based on the adherence to ribavirin [4,22]. The remaining studies analyzed the influencing factors on the basis of combined measures for ribavirin and interferon. In two studies adherence was operationalized as the mean proportion of doses taken [4,23]. In all other studies adherence was operationalized as the proportion of patients taking a certain number of doses (adherent patients). Besides two exceptions [8,27], the threshold for classifying patients as adherent was set to 80%. The characteristics of studies are presented in Table 2.

**Table 2:** Study characteristics

**Study**	**Study type**	**Number of patients**	**Observation period**	**Inclusion criteria**	**Setting/country**	**Adherence measurement**	**Adherence operationalization**	**Adherence rate of patients**
Giannelli 2012 [21]	Cohort study	342	48 week	Transplantation for hepatitis-C virus-related liver disease	Liver transplantation centre/Italy	NR	Patients taking ≥80% of doses (ribavirin and interferon)	0.38
				Transplanted for at least 6 months				
				Positive test for anti-hepatitis-C virus and hepatitis-C virus RNA				
				Liver biopsy demonstrating a recurrence of chronic hepatitis-C				
				Treated with ribavirin plus peg-interferon interferon				
				No coexistent hepatitis-B				
				No cirrhosis				
Lo Re [4]	Cohort study	5706	90 days	US veterans	Veterans affairs medical facilities/USA	Pill count	Doses taken (ribavirin)	0.86
				Hepatitis-C virus infected				
				Genotype 1, 2, 3, or 4				
				At least one prescription each for peg-interferon and ribavirin				
				Viral load prior to hepatitis-C virus therapy				
				Viral load after treatment initiation				
				Not HIV-infected				
Marcellin 2011 [8]	Cohort study	1510	6 month follow-up after end of treatment	Chronic hepatitis-C	University hospitals, non-University hospitals and private practice offices, hospitals/France	Self-report (doses taken)	Patients taking 100% of doses (peg-interferon alpha-2b and ribavirin)	0.38
				≥18 years				
				Initiating therapy				
				Therapy with peg-interferon alpha-2b and ribavirin				
Martín-Santos 2008 [22]	Cohort study	146	4-24 weeks	Chronic Hepatitis-C	Hospitals/Spain	NR	Patients taking ≥80% of doses (ribavirin)	0.89
				Therapy with peg-interferon alpha-2a and ribavirin				
				Substance abuse abstinence ≥ 6 months				
				No cognitive or language difficulties				
				No other liver diseases				
				No co-infection with hepatitis-B or HIV				
				No hepatocellular carcinoma				
				No autoimmune disorders				
				Neutrophil count f >1.5 × 10^9^⁄l				
				Platelet count of >75 × 10^9^⁄l				
				No psychiatric disorders other than affective disorders				
Rodis [23]	Cohort study	12	3 month	NR	Interdisciplinary HCV education and monitoring service/USA	Self-report (Morisky scale, Brief medication questionnaire)	Doses taken (interferon and ribavirin)	1.00
Sola [24]	Cohort study	157	72 weeks	Chronic Hepatitis-C and hepatitis-C virus RNA positive in plasma	Hospitals/Spain	Self-report via daily questionnaire (ribavirin and interferon) Pill counts (ribavirin)	Patients taking ≥80% of doses (ribavirin and interferon)	0.76
				≥18 years				
				Elevated alanin-aminotransferase levels				
				Findings on liver biopsy consistent with presence of chronic hepatitis-C , and compensated liver disease				
				CD4 cell count >200 × 10^6^/mm^3^ regardless of plasma HIV RNA level or CD4 cell count <200 × 10^6^/mm^3^ wiimes;th undetectable HIV RNA level				
				Neutrophil count ≥1500/mm^3^; platelet count ≥70,000/mm^3^; hemoglobin level ≥11 g/dl for women, or ≥12 g/dl for men				
				No previous treatment with interferon or ribavirin				
				No hepatitis-A or –B co-infection				
				No liver disease				
				No decompensated cirrhosis				
				No pregnancy				
				No active drug or alcohol consumption within the last 6 months				
				Potential contraindications to interferon or ribavirin				
Sylvestre [25]	Cohort study	71	48 weeks	Hepatitis-C	Clinic/USA	Self-report by monthly questionnaire Pill count	Patients taking >80% of doses (interferon alpha -2b and ribavirin)	0.68
				≥18 years				
				Maintained on methadone for 3 months				
				At least 75% attendance at our weekly clinics for a period of at least 2 months				
				No other liver disease				
				No untreated depression				
Tanioka [26]	Cohort study	363	8 months	Hepatitis-C	Hospitals/Japan	NR	Patients taking >80% of doses (interferon alpha -2b and ribavirin)	0.52
				≥18 years				
				Aminotransferase above the upper normal limit in the 6 months before entry in to the study				
				Compensated liver function with normal levels of serum albumin, prothrombin time and serum bilirubin				
				No chronic liver diseases				
				No injected drugs or abused alcohol within the previous 6 months				
				No poorly controlled psychiatric illness				
				Not HIV positive				
				No cirrhosis				
Wagner [27]	Cross-sectional study	72	NA	Hepatitis-C virus	Veterans administration	Self-report (VAS)	Patients taking 100% of doses (peg-interferon and ribavirin)	0.94
				HIV				
				Interferon based hepatitis-C virus treatment	Medical center/USA			

NA: not applicable; NR: not reported.

The measured adherence rates in the studies using the 80% threshold (seven out of nine studies) ranged from 0.38 to 0.94.

Out of the factors that were analyzed in only one study “anemia”, “new use of growth factors”, “new use of thyroid medications”,, “intended treatment duration”, “(actual) treatment duration”, “leucocyte count”, and “treatment center size” showed a statistically significant influence on adherence. The results of the individual analyses for each influencing factor are presented in Table 3.

**Table 3:** Influence of factors on adherence for factors that were analyzed in one study

**Study**	**Factor**	**Effect direction or compared categories; effect size; 95%-CI or p-value**
Giannelli 2012 [21]*	Anemia	Negative; NR; < 0.05
Lo Re [4]	Methadone use	Yes vs. no; OR = 0.99; 0.167
	New use of growth factors	Yes vs. no; OR = 1.01; 0.021
	New use of thyroid medication	Yes vs. no; OR = 1.02; 0.022
	Schizophrenia	Yes vs. no; OR = 1.00; 0.605
Marcellin 2011 [8]*	Adverse events	Yes vs. no; OR^#^ = 1.09; 0.77 to 1.54
	Diabetes (baseline and follow-up)	NR; NR; > 0.05
	Duration of infection	Positive; NE; 0.601^#^
	Fibrosis scores	NR; NR; > 0.05
	HCB positive	Yes vs. no; OR^#^ = 1.47; 0.62 to 3.48
	Naïve for Hepatitis-C virus treatment	Yes vs. no; OR = 1.32; 1.03 to 1.69
	Origin of incomes	Paid employment vs. others; OR^#^ =1.12; 0.91 to 1.38
	Other chronic disease (baseline)	Yes vs. no; OR^#^ = 0.91; 0.72 to 1.15
	Remoteness of the center (transport time)	Per min; OR = 1.00; 1.00 to 1.01
	Source of Hepatitis-C virus infection	Intra venous drug use vs. others; OR^#^ = 0.99; 0.80 to 1.22
	Therapeutic education (not specified)	NR; NR; > 0.05
Sola [24]*	Alanin-Aminotransferase	NR; NR; > 0.05
	Alkaline phosphatase	NR; NR; > 0.05
	Aspartate aminotransferase	NR; NR; > 0.05
	Fibrosis metavir score	NR; NR; > 0.05
	Leukocytes	Positive; NE; 0.007^#^
	Platelets	NR; NR; > 0.05
	Prothrombin time	NR; NR; > 0.05
	Serum albumin	NR; NR; > 0.05
	Serum bilirubin	NR; NR; > 0.05
Sylvestre [25]*^#^	Duration of abstinence	≥ 1 month vs. < 1 month; MD = 27%; 0.10
	Psychiatric medication (baseline)	NR; NR; 0.2
	Psychiatric medication (during treatment)	NR; NR; 0.3
	Psychiatric medication (initiation during treatment)	Yes vs. no; MD = -28%; 0.02
Tanioka [26]	Physicians experience (years)	≥ 19 vs. < 19; RR = 1.54; 0.96 to 2.48
	Platelet count (10^4^/ml)	≥ 15 vs. < 15; RR^#^ = 0.86; 0.57 to 1.29
	Treatment center size (cases per center)	≥ 15 vs. < 15; RR = 1.65; 1.04 to 2.64
	White blood cell count (ml)	≥ 5000 vs. < 5000; RR^#^ = 1.16; 0.77 to 1.75
Wagner [27]*^#^	Drinking problem	Yes vs. no; OR = 0.63; 0.16 to 2.44
	HIV RNA	≤ 400 vs. >400; OR = 1.83; 0.51 to 6.53
	In a relationship	Yes vs. no; OR = 0.81; 0.24 to 2.75

NE: not reported and not estimable (e.g. continuous variable); NR: not reported; OR: odds ratio; RR: relative risk; *Analysis based on combined adherence rates for interferon and ribavirin; ^#^univariate; wording according to publication.

18 influencing factors were analyzed in more than one study (age, alcohol consumption, depression, [illicit] drug use, education, employment status, ethnic group, gender, genotype, haemoglobin, hepatitis-C virus RNA, HIV co-infection, medication dose interferon, medication dose ribavirin, metavir activity, psychiatric disorder, treatment experience, weight). The factors that were analyzed in more than one study are presented in Table 4.

**Table 4:** Influence of factors on adherence for factors that were analyzed in more than one study

**Factor**	**Study**	**Effect on adherence (effect direction or compared categories; effect size; p-value)**
Age	Marcellin 2011 [8]*	NR ; NR; > 0.05
	Sola [24]*	NR; NE; 0.01^#^
	Sylvestre [25]*^#^ (years)	< 55 vs. ≥ 55; RR = 2.38; 1.49 to 3.80
	Tanioka [26]	NR; NR; 0.59
	Wagner [27]*^#^	NR; NR; 0.59
Alcohol consumption	Marcellin 2011 [8]* ≥20 g/day (baseline and follow-up)	NR; NR; > 0.05
	Sola [24]*	NR; NR; > 0.05
Depression	Lo Re [4] (bipolar/depression)	Yes vs. no; OR = 1.00; 0.945
	Martín-Santos 2008 [22] (depression and anxiety)^#^ [Patient Health Questionnaire]	Yes vs. no; MD = -13%; 0.02
	Rodis [23]	Positive associated with adherence indicators^†^
	Wagner [27]*^#^	Yes vs. no; OR = 0.48; 0.16 to 1.40
Drug use	Marcellin 2011 [8]*	Yes vs. No; OR = 0.42; 0.23 to 0.77
	Sola [24]*	NR; NR; > 0.05
	Sylvestre [25]*^#^ (regular cocaine/methamphetamin)	Yes vs. no; MD = -1%; > 0.05
		Yes vs. no; MD = -20%; 0.10
		Regular vs. none, rarely, intermittent; MD = -48%; 0.03
	Wagner [27]*^#^	Yes vs. no; OR = 1.42; 0.27 to 7.52
Education	Marcellin 2011 [8]* (not specified)	Low vs. high; OR^#^ = 0.99; 0.80 to 1.23
	Wagner [27]*^#^	Any college vs. others; OR = 1.75; 0.60 to 5.11
Employment status	Marcellin 2011* [8]	Unemployed vs. others; OR^#^ = 1.01; 0.75 to 1.37
	Wagner [27]*^#^	Yes vs. no; OR = 0.61; 0.20 to 1.81
Ethnic group	Sylvestre [25]*^#^	NR; NR; 0.2
	Wagner [27]*^#^	African American vs. others; OR = 0.50; 0.16 to 1.51
Gender	Giannelli [21]	Male vs. female; OR^#^ = 2.44; 1.41 to 4.22
	Marcellin 2011* [8]	NR; NR; > 0.05
	Sola [24]*	NR; NR; > 0.05
	Sylvestre [25]*^#^	NR; NR; 0.4
	Tanioka [26]	Male vs. female; RR = 2.01; 1.07 to 3.79
	Wagner [27]*^#^	Male vs. female; OR = 0.85; 0.08 to 8.74
Genotype	Marcellin 2011* [8]	3 vs. 1; OR = 1.55; 1.20 to 2.01
	Sola [24]*	1 or 4 vs. 2 or 3; OR = 2.6; 1.1 to 6.7
	Sylvestre [25]*^#^	2 or 3 (24 weeks treatment) vs. 1 (48 weeks treatment); MD = 23%; 0.07
	Tanioka [26]	2 vs. 1; RR = 1.84; 1.10 to 3.09
	Wagner [27]*^#^	1 or 4 vs. other; OR = 0.81; 0.25 to 2.66
Haemoglobin level	Sola [24]*	>14.9 g/dl vs. < 14.9 g/dl; OR = 3.3; 1.4 to 8.1
	Tanioka [26]	≥ 14 vs. < 14; RR = 1.50; 0.85 to 2.64
Hepatitis-C virus RNA	Marcellin 2011* [8]	NR; NR; > 0.05
	Sola [24]*	NR; NR; > 0.05
	Tanioka [26]	< 100 vs. ≥100; RR^#^ = 0.49; 0.18 to 1.34
	Wagner [27]*^#^	<800,000 vs. other; OR = 1.03; 0.35 to 3.01
HIV co-infection	Marcellin 2011* [8]	Yes vs. no; OR = 2.52; 1.36 to 4.67
	Sola [24]*	NR; NR; > 0.05
Medication dose interferon	Marcellin 201* [8] (Peg-Interferon)	Positive; NE; 0.003^#^
	Tanioka [26] (million units/kg)	< 0.13 vs. ≥ 0.13; RR = 2.42; 1.52 to 3.85
Medication dose ribavirin	Giannelli [21]	Negative; NR; < 0.05
	Marcellin 201* [8]	Negative; NE; 0.097^#^
	Tanioka [26] (mg/kg)	< 11 vs. ≥11; RR^#^ = 1.12; 0.75 to 1.81
Metavir activity	Marcellin 2011* [8]	NR; NR; > 0.05
	Sola [24]* (Score)	NR; NR; > 0.05
Psychiatric disorder	Lo Re [4]	Yes vs. no; OR = 0.99; 0.226
	Marcellin 2011 [8]* (baseline and follow up)	NR; NR; > 0.05
	Sylvestre [25]*^#^	Yes vs. no; MD = to -8%; > 0.05
	Wagner [27]*^#^	Yes vs. no; OR = 0.25; 0.08 to 0.76
Treatment experience	Lo Re [4] (duration)	Decline per 12 weeks; mean = 0.001
	Marcellin 2011 [8]* (intended duration)	Negative; NE; <0.001^#^
	Tanioka [26]	Retreatment vs. naive; RR = 1.86; 1.15 to 3.01
Weight	Marcellin 2011 [8]*	NR; NR; > 0.05
	Sola [24]*	NR; NR; > 0.05
	Tanioka [26] (kg/BMI)	≥ 60 vs. < 60; RR = 1.09; 0.63 to 1.89
		NR; RR^#^ = 1.11; 0.73 to 1.69

NE: not reported and not estimable (e.g. continuous variable); NR: not reported; OR: odds ratio; RR: relative risk; *Analysis based on combined adherence rates for interferon and ribavirin; ^#^univariate; ^**†**^wording according to publication.

“Depression” showed a mainly negative effect on adherence. Two out of four studies showed a statistically significant effect [4,8,23,27]. However, one study showed a conflicting effect direction [23].

Also “psychiatric disorders in general” found to have a negative effect on adherence. All studies that analyzed this factor showed this effect direction and one study was statistically significant [4,8,25,27].

Higher doses of ribavirin were associated with lower adherence. The effect direction between the studies was consistent and one study was statistically significant [8,21,26].

There is a strong tendency that having a “HIV co-infection” influences adherence positively. One out of two studies was statistically significant and showed a large effect size [8,24].

The “hemoglobin level” showed a positive effect on adherence. Both studies that analyzed this outcome showed this effect direction and one was statistically significant [24,26].

There is the tendency that male patients are more adherent than female patients. Two studies were statistically significant in favor of this effect direction. But one statistically non-significant study showed a conflicting effect direction and three studies were statistically not significant [8,21,24-27].

“Alcohol consumption” [8,24], “education” [8,27], “employment status” [8,27], “ethnic group“ [25,27], ”hepatitis-C virus RNA” [8,24,26,27], “genotype”, “metavir activity” [8] and “weight” [8,24,26], showed no statistically significant effect throughout. Although some studies showed statistically significant results for “age”, “drug use”, “genotype”, “medication dose interferon“, and “treatment experience” the effect is unclear because effect directions were partly conflicting i.e. some studies showed a positive effect and some studies a negative effect on adherence.

## Discussion

This is the first review that systematically analyzes adherence influencing factors in hepatitis-C infected patients taking ribavirin. There are several factors that seem to influence adherence in hepatitis-C infected patients taking ribavirin. “Psychiatric disorders/depression”, “higher doses ribavirin” seem to have a negative influence on adherence. In contrast “HIV co-infection” and “hemoglobin level” seem to have a positive influence on adherence. Furthermore, there is the tendency that male patients are more adherent than female patients. “Alcohol consumption”, “education”, “employment status”, “ethnic group“, ”hepatitis-C virus RNA”, “genotype”, “metavir activity” and “weight” seem to have no effect on adherence. The remaining the results differed between studies.

The findings are in accordance with research findings for other indications. A meta-analysis found a statistically significant negative effect of depression on adherence in chronic conditions [28]. This might be attributable to a reduced motivation in depressed patients. The question is therefore, whether the treatment of the psychiatric disorder can help to increase adherence.

The negative influence of higher doses ribavirin on adherence is probably caused by the higher risk of side effects. For example, systematic reviews in HIV infected patients have shown that side effects are a predictor for non-adherence [29,30]. The assumption that ribavirin intake can be associated with depression is justified. A low hemoglobin level is associated with fatigue which can possibly result in low motivation to take medication. Furthermore, also a low hemoglobin level and respectively the associated fatigue is a possible side effect of ribavirin. Therefore, the hemoglobin level is perhaps also an indicator for side effects.

The two studies that analyzed the influence of an HIV-co-infection are adjusted for drug use [8,24]. The reason why this confounder is adjusted for the positive effect of an HIV-co-infection might be due to the experience in handling complex treatment regimens in HIV-infected individuals. Furthermore research has indicated, that access to care is higher in co-infected individuals [31].

Due to the heterogeneity no general conclusions can be made that can be applied to all settings, countries, patient groups, etc. This pertains also for the factors that were highlighted as having an influence, The results should rather be considered explorative as indications for factors that can have an influence on adherence in hepatitis-C infected patients treated with regimes that contain ribavirin. To be of sufficient significance to make decisions in clinical practice, the factor/s has/have to be evaluated in detail for the specific context of the decision. The main reasons for heterogeneity between studies are the sample size, the analyses methods, different regimens and different patient characteristics. Furthermore, all studies revealed methodological flaws. In particular the measurement of influencing factors was mostly unclear. Also the time point of measurement can have an influence on adherence. A more recent study shows that at the first measurement time point younger age and African American ethnicity were statistically significant associated with lower ribavirin adherence. At the second measurement time point these factors were not statistically significant anymore, but publicly insured and employed patients showed a statistically significant effect in ribavirin adherence.

The measurement of adherence is performed with various instruments. All types of the applied adherence measurement instruments are associated with the tendency to overestimate adherence [32]. Most studies use self-reports. In particular for self-reporting instruments a higher estimation of intake rather than the true adherence rate has been shown [32]. Indeed pill counts and prescription refill are a more objective adherence measures but also these measurement methods imply the tendency to overestimate adherence (e.g. trashing tablets). In none of the included studies timing adherence was assessed. Thus, for example compensating one missing ribavirin tabled by double taking on another day would not have been revealed. However, for a more detailed and precise assessment usually additional effort is necessary which is often not feasible in clinical practice.

To have a substantial virologic response, patients have to reach a certain adherence level. Taking this into account, the proportion of patients reaching this cut-off value should be chosen as the operationalization of adherence, instead of the mean of the entire trial population, as the overall mean does not allow for a clinically significant estimation of how many patients can reach the required adherence. To our knowledge, a precise lower bound of required adherence (dose and timing) for an adequate suppression of RNA replication has not yet been proven [7]. Thus, the cut-off values used in the studies are not proven. It has to be taken into account that also the variation between patients and regimens should be analyzed in detail in this context because the needed adherence to reach a substantial virologic response probably depends on patient characteristics and/or the regimen. Furthermore, prior research has shown that a categorization of variables can result in different predictors in prognostic models and in poor performance of the model [33]. However, the mean adherence is only used as operationalization for adherence in two studies [4,23]. Apart from this, it is unlikely that adherence is influenced by only one factor but it is rather a multifactorial problem [9].

The different adherence operationalization and measurements are furthermore a limitation for the comparability of results and probably one reason for different results regarding the statistical significance and effect direction between studies. But also the influencing factors differ regarding operationalization and measurement. For example in all studies age is operationalized in two categories or continuously. However, studies on other indications have shown that adherence presents a concave shape i.e. adherence is highest in the middle age and declines with younger or older age [34]. Such information is lost (no statistically significant results) if e.g. only two categories are used or age is treated as a continuous variable. The effect of different categorizations for the same influencing factor on the results is analyzed in none of the included studies (sensitivity analysis).

Another comparability limiting point is that the analyses are adjusted for different factors. Especially the unadjusted analysis should be interpreted with caution because confounders or effect modifiers are not accounted for. But also the multivariate analyses are adjusted for different factors and consequently the comparability is limited. Although, it was sought to consider confounding in the evidence synthesis, i.e. to identify factors that are independently associated with adherence, a risk of bias in the results cannot be excluded.

In two studies, variables that do not contribute to the explanation of the variance of adherence were not eliminated from the analysis. Consequently the probability of statistically non-significant results due to inter-correlation might be raised [25,35]. In the other multivariate analyses indeed the model is fitted by eliminating variables without a statistically significant influence on adherence. However, in none of the multivariate analysis the inter-correlations (e.g. drug use and alcohol use) between influencing factors were analyzed. Thus, variables that measure basically the same phenomena (e.g. mental illness) probably show no influence in the analysis, because most of the variance in adherence is explained by one factor (e.g. drug use) leaving little potential for explaining additional variance in adherence by adding the other factor (e.g. alcohol use). The actual influencing factor or underlying phenomena can therefore be concealed. In addition some factors that have shown an influence in other conditions like copayments and other barriers to access to care were not analyzed in any of the included studies [36].

The observed high adherence rates in some studies suggest a “ceiling effect”. A high overall adherence level implies that adherence differences become marginal. Probably the high adherence is due to the fact that patients participating in studies are often more adherent than those patients, who refuse study participation [37]. Furthermore, it can be presumed that access to medication is ensured for study participants. The high baseline adherence implies that a large sample size is needed to show statistical significance of the results. However, most studies were small and thus probably underpowered.

The presented systematic review has some limitations. Firstly, missing relevant literature published in other languages could not be excluded because we included only English and German literature [38]. Secondly, we did not evaluate the quality of registry data in register based studies. The extent of this source of bias is therefore unknown. Thirdly, we did not evaluate the risk of bias for each individual factor, because in most studies for none of the factors the measurement was described in detail and consequently all factors would have had to be rated with unclear risk of bias. But an unclear risk of bias was judged differently depending on the factor in the evidence synthesis (e.g. age vs. social support).

In this systematic review only implementation adherence to antiretroviral hepatitis-C therapy was considered because, persistence and implementation adherence should been analyzed separately [15]. It could be hypothesized that early implementation non-adherence is associated with discontinuation. However, in a study that analyzed many various potential influencing factors only younger age showed an influence on discontinuation and also on ribavirin implementation adherence. Another study showed no statistically significant association between adherence and cannabis users, but cannabis users were statistically significant more likely to continuing treatment [25]. Also other studies indicate that the factors influencing implementation adherence and discontinuation differ. Thus, this systematic review indicates an association between depression and adherence [39]. Again, a study on the influence of depression on discontinuation in intravenous drug users found not statistically significant association. Another study showed a statistically non-significant influence of drug addiction and a non-significant effect of psychiatric deterioration on discontinuation [40]. Also these results were contrary to the presented results for implementation adherence.

In clinical practice the factors can be an indication for non-adherence, especially if various factors pertain in one patient. Due to the explorative nature of our analysis, adherence influencing factors in hepatitis-C infected patients receiving combination therapy with ribavirin should further be investigated to get deeper insights into the reasons for non-adherence. Detailed knowledge of adherence influencing factors would facilitate the identification of patients at risk for non-adherence e.g. the development of screening tools for non-adherence. The knowledge of adherence influencing factors can also contribute to the development of tailored, multifactorial adherence enhancing interventions.

## Conclusion

There are some factors that seem to show an influence on adherence. However, due to the heterogeneity (e.g. patient characteristics, regimes, settings, countries) no general conclusions can be made. The results should rather be considered as indications for factors that can have an influence on adherence in hepatitis-C infected patients taking regimes that containing ribavirin.

### Ethics statement

This is a systematic literature review an involves no human subjects human material or human data.

## Competing interests

No of the authors has anything to disclose. There was no funding for this review.

## Authors’ contributions

TM and DP: study concept and design; TM, DP, S-LA: acquisition of data; TM, DP: analysis and interpretation of data; TM: drafting of the manuscript: DP, S-LA: critical revision of the manuscript; TM, DP: statistical analysis. All authors read and approved the final manuscript.

## Pre-publication history

The pre-publication history for this paper can be accessed here:

http://www.biomedcentral.com/1471-2334/14/203/prepub

## Supplementary Material

Additional file 1 Search strategy. Click here for file

Additional file 2 Evaluation questions and ratings. Click here for file
