# Supplementary material for: Factors influencing adherence in Hepatitis-C infected patients: a systematic review
Source: BMC Infect Dis. 2014 Apr 15;14:203. doi: 10.1186/1471-2334-14-203 (PMC4021290; doi:10.1186/1471-2334-14-203)
Supplement: Additional file 1 — Search strategy. [file 1471-2334-14-203-S1.docx]

Supplement I: Search strategy

| **Database (provider)** | **Search strategy** |
| --- | --- |
| **MEDLINE (Pubmed)** | ("hepatitis C" [MeSH Terms] OR "hep C" [Title/Abstract] OR HCV [Title/Abstract] OR CHC [Title/Abstract] OR AHC [Title/Abstract] OR Hepacivirus [Title/Abstract]) AND (Adherence [Title/Abstract] OR adherent [Title/Abstract] OR adhere [Title/Abstract] OR nonadherence [Title/Abstract] OR nonadherent [Title/Abstract] OR Compliance [Title/Abstract] OR "patient compliance" [MeSH Terms] OR compliant [Title/Abstract] OR comply [Title/Abstract] OR noncompliance [Title/Abstract] OR noncompliant [Title/Abstract]) AND (ribavirin [MeSH Terms] OR ribavirin [Title/Abstract] OR) AND (risk factors [mesh] OR factor [TIAB] OR factors [TIAB] OR predict [TIAB] OR predictor [TIAB] OR predictors [TIAB] OR indicate [TIAB] OR indicator [TIAB] OR indicators [TIAB] OR influence [TIAB] OR influencing [TIAB] OR determinate [TIAB] OR determinates [TIAB] OR determination [TIAB] OR barrier [TIAB] OR barriers [TIAB] OR facilitate [TIAB] OR facilitator [TIAB] OR facilitators [TIAB]) AND (English [la] OR german [la]) |
| **Embase (Embase)** | (’hepatitis C’/exp OR ’hep C’:ti,ab OR HCV:ti,ab OR CHC:ti,ab OR AHC:ti,ab OR Hepacivirus:ti,ab) AND (Adherence:ab,ti OR adherent:ab,ti OR adhere:ab,ti OR nonadherence:ab,ti OR nonadherent:ab,ti OR Compliance:ab,ti OR ‘patient compliance'/exp OR compliant:ab,ti OR comply:ab,ti OR noncompliance:ab,ti OR noncompliant:ab,ti) AND ('risk factor'/exp OR factor:ab,ti OR factors:ab,ti OR predict:ab,ti OR predictor:ab,ti OR predictors:ab,ti OR indicate:ab,ti OR indicator:ab,ti OR indicators:ab,ti OR influence:ab,ti OR influencing:ab,ti OR determinate:ab,ti OR determinates:ab,ti OR determination:ab,ti OR barrier:ab,ti OR barriers:ab,ti OR facilitate:ab,ti OR facilitator:ab,ti OR facilitators:ab,ti OR hindrance:ab,ti OR hindrances:ab,ti) AND (ribavirin/exp) AND (English OR german):la AND 'article'/it |
