# Supplementary material for: Factors influencing adherence in Hepatitis-C infected patients: a systematic review
Source: BMC Infect Dis. 2014 Apr 15;14:203. doi: 10.1186/1471-2334-14-203 (PMC4021290; doi:10.1186/1471-2334-14-203)
Supplement: Additional file 2 — Evaluation questions and ratings. [file 1471-2334-14-203-S2.docx]

Supplement II: Evaluation questions and ratings

| 1. The study sample represents the population of interest with regard to key characteristics, sufficient to limit potential bias to the results |
| --- |
|  |
| 1. Loss to follow-up is unrelated to key characteristics (that is, the study data adequately represent the sample), sufficient to limit potential bias |
|  |
| 1. The prognostic factor of interest is adequately measured in study participants, sufficient to limit potential bias |
|  |
| 1. The outcome of interest is adequately measured in study participants, sufficient to limit bias |
|  |
| 1. Important potential confounders are appropriately accounted for, limiting potential bias with respect to the prognostic factor of interest |
|  |
| 1. The statistical analysis is appropriate for the design of the study, limiting potential for the presentation of invalid results |
|  |

Checklist items are worded so that a ‘yes’ response always indicates that the study has been designed and conducted in such a way as to minimize the risk of bias for that item. An ‘unclear’ response to a question may arise when the answer to an item is not reported or is not reported clearly.
